# Improving Observability of an Inertial System by Rotary Motions of an IMU

**DOI:** 10.3390/s17040698

**Published:** 2017-03-28

**Authors:** Shuang Du, Wei Sun, Yang Gao

**Affiliations:** 1 School of Aeronautics and Astronautics, University of Electronic Science and Technology of China, Chengdu 610051, China; 2 School of Geomatics, Liaoning Technical University, Fuxin 123000, China; sunwei-3775235@163.com; 3 Department of Geomatics Engineering, the University of Calgary, Calgary, AB T2N 1N4, Canada; ygao@ucalgary.ca

**Keywords:** inertial navigation system, MEMS IMU, observability, rotary motions

## Abstract

It has been identified that the inertial system is not a completely observable system in the absence of maneuvers. Although the velocity errors and the accelerometer bias in the vertical direction can be solely observable, other error states, including the attitude errors, the accelerometer biases in the east and north directions, and the gyro biases, are just jointly observable states with velocity measurements, which degrades the estimation accuracy of these error states. This paper proposes an innovative method to improve the system observability for a Micro-Electro-Mechanical-System (MEMS)-based Inertial Navigation System (INS) in the absence of maneuvers by rotary motions of the Inertial Measurement Unit (IMU). Three IMU rotation schemes, namely IMU continuous rotation about the X, Y and Z axes are employed. The observability is analyzed for the rotating system with a control-theoretic approach, and tests are also conducted based on a turntable to verify the improvements on the system observability by IMU rotations. Both theoretical analysis and the results indicate that the system observability is improved by proposed IMU rotations, the roll and pitch errors, the accelerometer biases in the east and north directions, the gyro biases become observable states in the absence of vehicle maneuvers. Although the azimuth error is still unobservable, the enhanced estimability of the gyro bias in the vertical direction can effectively mitigate the azimuth error accumulation.

## 1. Introduction

An inertial navigation system (INS) measures the specific forces and angular rates using a triad of accelerometers and gyroscopes (gyro) to determine the motion of a body with respect to the inertial frame. As a self-contained navigation system, INS has been used for a wide range of applications. For geomatics applications, INS is an essential geo-referencing device in mobile mapping systems for infrastructure and street surveys [1,2,3], in underwater navigation systems for offshore geophysical exploration and in unmanned aerial vehicles for disaster monitoring [4,5,6], just to mention a few. With advances in Micro-Electro-Mechanical Systems (MEMS), the chip-based inertial sensors are also increasingly adopted in place of traditional inertial systems for above applications because the MEMS inertial measurement units (IMU) are small-size, light-weight, low-power and low-cost although they feature significant sensor errors [7,8,9,10,11].

INS navigation errors include errors in its position, velocity and attitude solutions. Due to their self-contained characteristic, the INS navigation errors accumulate over time. For a MEMS-based inertial system, in particular, the navigation errors would accumulate quickly to a level of several kilometers within minutes due to significant inertial sensor errors [12,13]. In order to limit the navigation error accumulation, external measurements are often applied to estimate the navigation errors as well as inertial sensor errors using an extended Kalman filter (EKF) [14,15,16]. The most commonly used external measurements include position and velocity information from other sensors, such as Global Navigation Satellite System (GNSS) [13,17,18]. Since the position information is only weakly related to the attitude and inertial sensor errors, the velocity information then becomes the main measurements to estimate all other error parameters.

Research has been conducted to study the observability of the aided inertial systems. The control-theoretic approach was first proposed to analyze the observability of a time-varying system which can be modelled as the piece-wise constant system (PWCS) in [19], and the observability matrix was also developed in both the continuous and discrete representations for the observability analysis of such systems. Later, such approaches were applied to the inertial system and obtain the following findings [20]: (1) nine error states and linear combination of states are observable in the absence of maneuver, which are the three velocity errors and the accelerometer bias in the vertical direction, two linear combinations of attitude errors and the horizontal accelerometer biases, three linear combinations of attitude errors and gyro biases; (2) the observability is enhanced by maneuvering that all error states become observable when the changes in accelerations are present. The effect of different types of maneuvers on system observability was also investigated [21,22,23]. It was proved that although the turn maneuvers can improve the system observability, the constant accelerations without rotational motion cannot increase the number of observable states. Although the observability analysis was conducted in different aspects in the aforementioned researches, one common conclusion is that the system observability is poor in the absence of vehicle maneuvers. Except for the velocity errors and accelerometer bias in vertical direction, other errors cannot be uniquely estimated with the velocity measurements. The inertial system observability was also reviewed from the analytic estimation algorithm of the errors states in [24], which also addressed the differences between the “observability” and “estimability”, and the later one was analyzed based on the eigenvalues and eigenvectors of the covariance matrix.

Poor system observability would result in inaccurate estimation of the INS errors and eventually degrade the navigation performance. For instance, in precision agriculture, the GNSS/INS integrated system is employed to determine the position, velocity and attitude of the tractors for guidance and steering control. Parallel straight lines are typical for ploughing and cultivating with tractors [25]. When tractors travel at a constant velocity along straight lines, the vehicle maneuvers become very weak in such case. As a result, the observability for filter parameters of attitude errors, accelerometer biases in horizontal plane, and gyro bias in the vertical direction are very poor. In particular, the azimuth solution will be drifted away from its true value due to poor observability for the azimuth error and the gyro bias in the vertical direction. Similar conclusions are also indicated in [26], which shows that the azimuth and its rate gyro bias are unobservable in straight motion and with constant acceleration for land vehicle applications. The observability issue was also reported in underwater and aerial navigations [27,28]. Due to the water resistance, maneuvers are hard to be obtained for autonomous underwater vehicles (AUV), which also leads to the poor observability of azimuth error and its rate gyro bias [27]. For the helicopter unmanned aerial vehicle (UAV), the GPS-aided inertial navigation system (INS) does not provide the observability of the azimuth during hover [28].

To overcome the observability issues, additional sensors or external observations are usually employed. The tri-axial magnetometer was augmented to the navigation system to provide the heading observations for the UAV during hover in [28], and the GNSS-derived course angle was used as the additional observations to improve the observability in [29]. Furthermore, Wagner used the multi-antenna technique in automatic landing applications, and discussed the effects from different antenna configurations, aiding modes, as well as the lever arm imposed on the system observability [30]. Other than the aforementioned researches, the multi-position alignment procedure was designed for the vehicular fiber inertial system in [31,32], and simulations are conducted to verify that the collected additional observations with IMU rotated to other positions can improve the observability and alignment accuracy.

A few studies have proposed to use the IMU rotation to improve the system observability. The two most representative researches are given as follows. A calibration approach was designed for the INS with high-end IMUs by using the rotation of IMU about the azimuth axis in [33]. The sensor biases and attitude errors are estimated with external velocity and position measurements and the Singular Value Decomposition (SVD) was utilized to analysis the system observability. Simulation results indicate that although IMU rotation improves the system observability, the gyro bias in the vertical direction is still poor. The observability of INS with IMU rotation about the azimuth axis was also investigated based on the states’ covariance matrix of the filter to obtain the optimal rotation rate in [34]. The aforementioned references mainly employed the rotation to improve the system observability of INS during the alignment and calibration processes, however, the observability of the error states has not been revealed from the analytic point of view yet. Moreover, the following aspects of improving system observability by rotating IMU have not been discussed yet:

(1) The observability analysis for the low-cost MEMS-based INS: the existing researches are conducted only for high-end IMU cases. For the low-cost MEMS IMU, sensor errors are orders of magnitudes greater, comparing to the high-end IMUs, which will greatly affect the system observability.
(2) The performances evaluation on all axes IMU rotations: the previous work only involves the azimuth axis rotation, whereas the other two axes rotations lead to different observability results, which will be discussed in Section 3.
(3) The system observability for a 12-error state INS: Usually only 10 error states (horizontal velocity errors and accelerometer biases, as well as 3-dimensional attitude errors and gyro biases) are considered in the observability analysis [31,32,33,34], however, the velocity error and accelerometer bias in the vertical directions are also crucial for the applications of AUV and UAV navigation. Therefore, the observability of the 12-error state INS needs to be examined.

**Figure 1:**
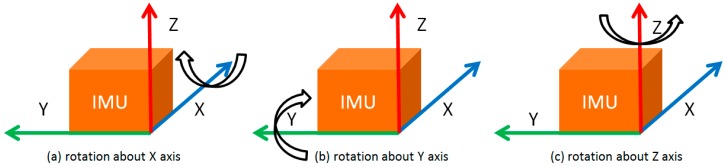
IMU rotation about the X, Y or Z axis.

This paper explores the observability of a MEMS-based inertial system with respect to the aforementioned aspects. Three single-axis rotations of IMU about the X, Y and Z axis are proposed as shown in Figure 1. Based on the observability definitions in [19,20], the observability of the inertial system with a rotating low-cost MEMS IMU is studied with a control-theoretic approach from the analytic point of view. Tests are also conducted using a tri-axial rotation table to verify the observability improvements by IMU rotations. Both the theoretical analysis and the results indicate that all error states, except for the azimuth error, become observable with IMU rotations about the X, Y, or Z axis, respectively. Although the azimuth error is still unobservable, the enhanced estimability of the gyro bias in the vertical direction can effective mitigate the azimuth error accumulation. The proper rotation of IMU can modulate the constant inertial bias into periodic signals and an integration of the modulated inertial data over a complete rotation cycle can eliminate the bias impact on the navigation solutions. More details regarding to this can be found in [12,35].

The remainder of this paper is organized as follows: Section 2 describes the error model for the MEMS-based inertial systems with both non-rotating and rotating IMUs, and Section 3 introduces the observability analysis for the non-rotating and rotating systems based on a control-theoretic approach. Section 4 presents the turntable tests and the corresponding results analysis, and the conclusions are summarized in Section 5.

## 2. Navigation Error Model for MEMS-Based INS

The error states for an inertial system usually include the position errors, the velocity errors, the attitude errors, and the accelerometer and gyro biases. For an inertial system in the absence of maneuvers, such as the vehicle remains still or travelling at constant velocity, the error behavior of velocity and attitude states in the navigation frame can be described by Equations (1)–(6). As the position errors are weakly related to the attitude errors and sensor errors, it is not considered in the observability analysis [36]:

(1) $$\delta {\dot{v}}_{E}=2\omega_{ie}\sin L\delta v_{N}-2\omega_{ie}\cos L\delta v_{U}+g\varepsilon_{N}+\gamma_{E}$$

(2) $$\delta {\dot{v}}_{N}=-2\omega_{ie}\sin L\delta v_{E}-g\varepsilon_{E}+\gamma_{N}$$

(3) $$\delta {\dot{v}}_{U}=2\omega_{ie}\cos L\delta v_{E}+\gamma_{U}$$

(4) $${\dot{\varepsilon }}_{E}=\omega_{ie}\sin L\varepsilon_{N}-\omega_{ie}\cos L\varepsilon_{U}+d_{E}$$

(5) $${\dot{\varepsilon }}_{N}=-\omega_{ie}\sin L\varepsilon_{E}+d_{N}$$

(6) $${\dot{\varepsilon }}_{U}=\omega_{ie}\cos L\varepsilon_{E}+d_{U}$$

where $\delta v_{E}$, $\delta v_{N}$, $\delta v_{U}$ are the velocity errors in the east, north and up directions, respectively; $\varepsilon_{E}$, $\varepsilon_{N}$, $\varepsilon_{U}$ are the attitude errors in the east, north and up directions, respectively; $\gamma_{E}$, $\gamma_{N}$, $\gamma_{U}$ are the accelerometer biases in the east, north and up directions, respectively; and $d_{E}$, $d_{N}$, $d_{U}$ are the gyro biases in the east, north and up directions, respectively; *L* is the latitude; $\omega_{ie}$ is the earth rotation rate.

For the low cost MEMS IMUs, the gyro biases are much greater than other terms in Equations (4)–(6), therefore, the error model for an MEMS-based INS can be simplified as described in Equation (7). The sensor biases are modeled as constants for observability analysis [20,21,22,23].

(7) $$\left[\begin{array}{c} \delta {\dot{v}}^{n}\\ {\dot{\varepsilon }}^{n}\\ {\dot{\gamma }}^{n}\\ {\dot{d}}^{n} \end{array}\right]=\left[\begin{array}{cccc} \Omega_{v} & F_{n} & I_{3\times 3} & 0\\ 0 & 0 & 0 & I_{3\times 3}\\ 0 & 0 & 0 & 0\\ 0 & 0 & 0 & 0 \end{array}\right]\left[\begin{array}{c} \delta v^{n}\\ \varepsilon^{n}\\ \gamma^{n}\\ d^{n} \end{array}\right]$$

where the superscript n represents the navigation frame; $\delta v^{n}={\left[\begin{array}{ccc} \delta v_{E} & \delta v_{N} & \delta v_{U} \end{array}\right]}^{T}$ is the velocity errors in the navigation frame; $\varepsilon^{n}={\left[\begin{array}{ccc} \varepsilon_{E} & \varepsilon_{N} & \varepsilon_{U} \end{array}\right]}^{T}$ is the attitude errors in the navigation frame, $\gamma^{n}={\left[\begin{array}{ccc} \gamma_{E} & \gamma_{N} & \gamma_{U} \end{array}\right]}^{T}$ is the accelerometer biases in the navigation frame; $d^{n}={\left[\begin{array}{ccc} d_{E} & d_{N} & d_{U} \end{array}\right]}^{T}$ is the gyro biases in the navigation frame; $\Omega_{v}$ describes the relationship between the velocity errors and their first time derivatives, it can be expressed by $2\omega_{e}\left[\begin{array}{ccc} 0 & \sin L & -\cos L\\ -\sin L & 0 & 0\\ \cos L & 0 & 0 \end{array}\right]$; $F_{n}$ is a 3 × 3 matrix, which describes the relationship between the attitude errors and the first time derivative of velocity errors, it can be expressed by $\left[\begin{array}{ccc} 0 & g & 0\\ -g & 0 & 0\\ 0 & 0 & 0 \end{array}\right]$, and g is the local gravity.

For an inertial system with a rotating IMU, the sensor frame, whose axes are aligned to the sensitive axis of IMU, is introduced. Although the sensor biases are modeled as constants in the sensor frame, these errors in the navigation frame become time-varying signals because of IMU rotations as shown in Equations (8) and (9), which calculates the sensor biases in the navigation frame and their first time derivatives, respectively. As the rotation of IMU does not introduce any linear movement, the Equations (1)–(6) also describe the velocity and attitude error behavior for an inertial system with a rotating IMU. Therefore, the error model for such a system can be described by Equation (10).

(8) $$\nabla^{n}=C_{b}^{n}C_{s}^{b}\nabla^{s}$$

(9) $${\dot{\nabla }}^{n}=C_{b}^{n}{\dot{C}}_{s}^{b}\nabla^{s}=C_{b}^{n}\Omega_{bs}^{b}C_{n}^{b}\nabla^{n}=R\nabla^{n}$$

(10) $$\left[\begin{array}{c} \delta {\dot{v}}^{n}\\ {\dot{\varepsilon }}^{n}\\ {\dot{\gamma }}^{n}\\ {\dot{d}}^{n} \end{array}\right]=\left[\begin{array}{cccc} \Omega_{v} & F_{n} & I & 0\\ 0 & 0 & 0 & I\\ 0 & 0 & R & 0\\ 0 & 0 & 0 & R \end{array}\right]\left[\begin{array}{c} \delta v^{n}\\ \varepsilon^{n}\\ \gamma^{n}\\ d^{n} \end{array}\right]$$

where ∇ represents the sensor (both accelerometer and gyro) biases, $C_{b}^{n}$ is the transformation matrix from the body frame to the navigation frame, it is also functions of roll, pitch and azimuth, $C_{s}^{b}$ is the transformation matrix from the sensor frame to the body frame, $\Omega_{bs}^{b}$ is the skew-symmetric matrix of $\omega_{bs}^{b}$, which is the rotation rate from the sensor frame to the body frame, expressed in the body frame, $\omega_{bs}^{b}$ equals to ${\left[\begin{array}{ccc} \omega & 0 & 0 \end{array}\right]}^{T},{\left[\begin{array}{ccc} 0 & \omega & 0 \end{array}\right]}^{T},{\left[\begin{array}{ccc} 0 & 0 & \omega \end{array}\right]}^{T}$ for IMU rotation about X, Y and Z axes, respectively.

## 3. Observability Analysis of MEMS-Based INS

The observability analysis approach for the linear system used in this paper is introduced as follows. Consider the linear system Σ described in Equation (11):

(11) $$\Sigma :\begin{array}{c} \dot{x}(t)=F(t)x(t)\\ z(t)=H(t)x(t) \end{array}$$

where x(t) and z(t) represent the system states and measurements, respectively, F(t) and H(t) are, respectively n × n and p × n matrices, n is the number of system states and p is the number of measurements. If F(t) and H(t) are constant, then Σ is a time-invariant linear system, and the time derivatives of the measurements can be described by Equation (12):

(12) $$\left[\begin{array}{c} z(t)\\ \dot{z}(t)\\ \ddot{z}(t)\\ \vdots \\ \overset{\left(n-1\right)}{z(t)} \end{array}\right]=\left[\begin{array}{c} H(t)x(t)\\ H(t)F(t)x(t)\\ H(t)F(t{)}^{2}x(t)\\ \vdots \\ H(t)F(t{)}^{n-1}x(t) \end{array}\right]=\left[\begin{array}{c} H(t)\\ H(t)F(t)\\ H(t)F(t{)}^{2}\\ \vdots \\ H(t)F(t{)}^{n-1} \end{array}\right]x(t)$$

According to [19], Σ is observable if and only if the rank of the matrix Q described in Equation (13) is *n*:

(13) $$Q=\left[\begin{array}{c} Q_{1}\\ Q_{2}\\ \vdots \\ Q_{k} \end{array}\right]=\left[\begin{array}{c} H(t)\\ H(t)F(t)\\ \vdots \\ H(t)F(t{)}^{n-1} \end{array}\right]$$

where $Q_{i}$ is the design matrix corresponding to the $i-1^{th}$ time derivative of measurements z(t).

For the inertial system, x(t) is the INS error state vector, including the velocity and attitude errors, as well as the accelerometer and gyro biases, F(t) is the INS error dynamic model, and H is a constant matrix and takes the form of $\left[\begin{array}{cccc} I_{3\times 3} & 0_{3\times 3} & 0_{3\times 3} & 0_{3\times 3} \end{array}\right]$ with velocity measurements. According to the error model described by Equation (7), the inertial system with a non-rotating IMU is time-invariant in the absence of maneuvers as F is constant matrix. Similarly, the system with a rotating IMU is also time-invariant, as long as the IMU rotation rate is constant according to Equations (9) and (10). Therefore, the observability analysis can be conducted based on above observability definitions for the MEMS-based INS with a non-rotating or rotating IMU, respectively. Our aim is to: (1) find the rank of Q matrix in Equation (13) for the non-rotating and rotating systems; (2) find the observable states or combinations of states based on Equation (12) for the non-rotating and rotating systems if the rank of Q matrix is smaller than n.

### 3.1. Observability of MEMS-Based INS with a Non-Rotating IMU

Based on the observability definition, the Q matrix is calculated as shown in Equation (14):

(14) $$Q=\left[\begin{array}{cccc} I_{3\times 3} & 0 & 0 & 0\\ \Omega_{v} & F_{n} & I_{3\times 3} & 0\\ {\Omega_{v}}^{2} & \Omega_{v}F_{n} & \Omega_{v} & F_{n}\\ {\Omega_{v}}^{3} & {\Omega_{v}}^{2}F_{n} & {\Omega_{v}}^{2} & \Omega_{v}F_{n}\\ \vdots & \vdots & \vdots & \vdots \\ {\Omega_{v}}^{k-1} & \sum_{i=1}^{k-1}{\Omega_{v}}^{k-1-i}F_{n} & {\Omega_{v}}^{k-2} & \sum_{i=1}^{k-2}{\Omega_{v}}^{k-2-i}F_{n} \end{array}\right]$$

**Property** **1.** *In the absence of maneuvers, the rank of* Q *is* *8, and the observable states are* $\delta v_{E}$*,* $\delta v_{N}$*,* $\delta v_{U}$*,* $\gamma_{U}$*,* $g\varepsilon_{N}+\gamma_{E}$*,* $-g\varepsilon_{E}+\gamma_{N}$*,* $d_{E}$ *and* $d_{N}$*.*

**Proof** **1.** Define the transformation

$$\Gamma =\left[\begin{array}{ccccc} I_{3\times 3} & 0 & 0 & 0 & 0\\ -\Omega_{v} & I_{3\times 3} & 0 & 0 & 0\\ 0 & -\Omega_{v} & I_{3\times 3} & 0 & 0\\ \vdots & \vdots & \vdots & \ddots & \vdots \\ 0 & 0 & 0 & -\Omega_{v} & I_{3\times 3} \end{array}\right]$$

and apply it to Q matrix, we can obtain the following equation:

(15) $$Q_{T}=\Gamma Q=\left[\begin{array}{cccc} I_{3\times 3} & 0 & 0 & 0\\ 0 & F_{n} & I_{3\times 3} & 0\\ 0 & 0 & 0 & F_{n}\\ 0 & 0 & 0 & 0\\ M & M & M & M\\ 0 & 0 & 0 & 0 \end{array}\right]=\left[\begin{array}{c} {Ν}_{1}\\ {Ν}_{2}\\ M\\ {Ν}_{k} \end{array}\right]$$

where ${Ν}_{i}$ is the design matrix corresponding to the transformed $i-1^{th}$ time derivative of measurements z(t). As the transformation Γ will not change the rank of the matrix, the rank of Q is same as the rank of $Q_{T}$, which is equal to the rank of ${\left[\begin{array}{ccc} {Ν}_{1} & {Ν}_{2} & {Ν}_{3} \end{array}\right]}^{T}$. By examining $F_{n}$, we can obtain that the rank of $Q_{T}$ is 8, which indicates there are 8 observable states or state combinations. According to [19,20], the observable states or states combinations can be obtained by $Q_{T}x(t)$ as shown in Equation (16).

(16) $$Q_{T}x(t)=\left[\begin{array}{c} {Ν}_{1}x(t)\\ {Ν}_{2}x(t)\\ {Ν}_{3}x(t)\\ 0_{3\times 1}\\ \vdots \end{array}\right]=\left[\begin{array}{c} \delta v^{n}\\ F_{n}\varepsilon^{n}+\gamma^{n}\\ F_{n}d^{n}\\ 0_{3\times 1}\\ \vdots \end{array}\right]=\left[\begin{array}{c} \delta v_{E}\\ \delta v_{N}\\ \delta v_{U}\\ g\varepsilon_{N}+\gamma_{E}\\ -g\varepsilon_{E}+\gamma_{N}\\ \gamma_{U}\\ d_{E}\\ d_{N}\\ 0\\ \vdots \end{array}\right]$$

The error states $\delta v_{E}$, $\delta v_{N}$ and $\delta v_{U}$ can be determined from ${Ν}_{1}x(t)$, then the linear combinations of error states, $g\varepsilon_{N}+\gamma_{E}$, $-g\varepsilon_{E}+\gamma_{N}$, and the error state, $\gamma_{U}$, can be obtained from ${Ν}_{2}x(t)$, and finally the gyro biases, $d_{E}$ and $d_{N}$ also become the observable based on ${Ν}_{3}x(t)$. Apparently, more measurement epochs neither increase the rank of $Q_{T}$ nor improve the system observability. The gyro bias in the up direction and the azimuth errors are unobservable without maneuvers. □

### 3.2. Observability of MEMS-Based INS with a Rotating IMU

For the system with a rotating IMU, the Q matrix can be calculated as shown in Equation (17):

(17) $$Q=\left[\begin{array}{cccc} I & 0 & 0 & 0\\ \Omega_{v} & F_{n} & I & 0\\ {\Omega_{v}}^{2} & \Omega_{v}F_{n} & \Omega_{v}+R & F_{n}\\ \vdots & \vdots & \vdots & \vdots \\ {\Omega_{v}}^{k-1} & \sum_{i=0}^{k-2}{\Omega_{v}}^{k-1-i}F_{n} & \sum_{i=0}^{k-2}{\Omega_{v}}^{k-1-i}R^{i} & \sum_{i=0}^{k-3}{\Omega_{v}}^{k-1-i}F_{n}R^{i} \end{array}\right]$$

By applying the transformation Γ, we can obtain $Q_{T}$ as shown in Equation (18):

(18) $$Q_{T}=\Gamma Q=\left[\begin{array}{cccc} I & 0 & 0 & 0\\ 0 & F_{n} & I & 0\\ 0 & 0 & R & F_{n}\\ 0 & 0 & R^{2} & F_{n}R\\ \vdots & \vdots & \vdots & \vdots \\ 0 & 0 & R^{k-2} & F_{n}R^{k-3} \end{array}\right]=\left[\begin{array}{c} {Ν}_{1}\\ {Ν}_{2}\\ \vdots \\ {Ν}_{k} \end{array}\right]$$

where ${\text{Ν}}_{i}$ is the design matrix corresponding to the transformed $i-1^{th}$ time derivative of measurements z(t).

**Property** **2.** *The rank of* $Q_{T}$ *is same as the rank of* $Q_{sub}={\left[\begin{array}{ccccc} {Ν}_{1} & {Ν}_{2} & {Ν}_{3} & {Ν}_{4} & {Ν}_{5} \end{array}\right]}^{T}$*.*

**Proof** **2.** According Equation (9), we can obtain the relationship between $R^{3}$ and R for IMU rotation about X, Y or Z axis, as follows.

(19) $$R^{3}=C_{b}^{n}{\left(\Omega_{bs}^{b}\right)}^{3}C_{n}^{b}=-\omega^{2}C_{b}^{n}\Omega_{bs}^{b}C_{n}^{b}=-\omega^{2}R$$

where ω is the IMU rotation rate. Then, for the matrix $Q_{T}$, we have that $\omega^{2}{Ν}_{i}+{Ν}_{i+2}=0_{3\times 12}$ when i > 3. Therefore, $Q_{T}$ can be transformed to ${\left[\begin{array}{ccccccc} {Ν}_{1} & {Ν}_{2} & {Ν}_{3} & {Ν}_{4} & {Ν}_{5} & 0_{3\times 12} & \cdots \end{array}\right]}^{T}$. Obviously that the rank of $Q_{T}$ is equal to the rank of $Q_{sub}={\left[\begin{array}{ccccc} {Ν}_{1} & {Ν}_{2} & {Ν}_{3} & {Ν}_{4} & {Ν}_{5} \end{array}\right]}^{T}$.

By examining $Q_{sub}x(t)$ shown in Equation (20), we can see that (1) based on ${Ν}_{1}x(t)$ and ${Ν}_{2}x(t)$, the velocity errors and accelerometer bias in the up direction are observable states, while the roll and pitch errors are jointly observable with accelerometer biases in east and north directions; (2) R and $R^{2}$ determine the rank of $Q_{sub}$ and other observable states:

(20) $$Q_{sub}x(t)=\left[\begin{array}{c} {Ν}_{1}x(t)\\ {Ν}_{2}x(t)\\ {Ν}_{3}x(t)\\ {Ν}_{4}x(t)\\ {Ν}_{5}x(t) \end{array}\right]=\left[\begin{array}{c} \delta v^{n}\\ F_{n}\varepsilon^{n}+\gamma^{n}\\ R\gamma^{n}+F_{n}d^{n}\\ R^{2}\gamma^{n}+F_{n}Rd^{n}\\ -\omega^{2}R\gamma^{n}+F_{n}R^{2}d^{n} \end{array}\right]$$

As R and $R^{2}$ are related to the IMU rotations, the following observability analysis is conducted w.r.t. IMU rotation about X, Y and Z axis, respectively. □

(1) Continuous Rotation about X axis

**Property** **3.** *When roll is zero, the rank of* $Q_{sub}$ *is 10, and the observable states or state combinations are:* $\delta v_{E}$*,* $\delta v_{N}$*,* $\delta v_{U}$*,* $g\varepsilon_{N}+\gamma_{E}$*,* $-g\varepsilon_{E}+\gamma_{N}$*,* $\sin A\gamma_{E}+\cos A\gamma_{N}$*,* $\gamma_{U}$*,* $d_{E}$*,* $d_{N}$*,* $d_{U}$*; when roll is not zero, the rank of* $Q_{sub}$ *is 11, and the following states can be uniquely determined:* $\delta v_{E}$*,* $\delta v_{N}$*,* $\delta v_{U}$*,* $\varepsilon_{E}$*,* $\varepsilon_{N}$*,* $\gamma_{E}$*,* $\gamma_{N}$*,* $\gamma_{U}$*,* $d_{E}$*,* $d_{N}$*,* $d_{U}$.

**Proof** **3.** When roll is zero, R and $R^{2}$ are calculated as shown in Equations (21) and (22), respectively, and ${\left[\begin{array}{ccc} {Ν}_{3} & {Ν}_{4} & {Ν}_{5} \end{array}\right]}^{T}x(t)$ can be calculated as shown in Equation (23)

(21) $$R=C_{b}^{n}\Omega_{bs}^{b}C_{n}^{b}=\omega \left[\begin{array}{ccc} 0 & 0 & -\sin A\\ 0 & 0 & -\cos A\\ \sin A & \cos A & 0 \end{array}\right]$$

(22) $$R^{2}=C_{b}^{n}\Omega_{bs}^{b}\Omega_{bs}^{b}C_{n}^{b}=\omega^{2}\left[\begin{array}{ccc} -\sin^{2}A & -\sin A\cos A & 0\\ -\sin A\cos A & -\cos^{2}A & 0\\ 0 & 0 & -1 \end{array}\right]$$

(23) $$\left[\begin{array}{c} {Ν}_{3}x(t)\\ {Ν}_{4}x(t)\\ {Ν}_{5}x(t) \end{array}\right]=\left[\begin{array}{c} -\omega \sin A\gamma_{U}+gd_{N}\\ -\omega \cos A\gamma_{U}-gd_{E}\\ \omega (\sin A\gamma_{E}+\cos A\gamma_{N})\\ -\omega^{2}\sin A(\sin A\gamma_{E}+\cos A\gamma_{N})-g\omega \cos Ad_{U}\\ -\omega^{2}\cos A(\sin A\gamma_{E}+\cos A\gamma_{N})+g\omega \sin Ad_{U}\\ -\omega^{2}\gamma_{U}\\ \omega^{3}\sin A\gamma_{U}-g\omega^{2}\cos A(\sin Ad_{E}+\cos Ad_{N})\\ \omega^{3}\cos A\gamma_{U}+g\omega^{2}\sin A(\sin Ad_{E}+\cos Ad_{N})\\ -\omega^{3}(\sin A\gamma_{E}+\cos A\gamma_{N}) \end{array}\right]$$

where *A* is the azimuth. As $\gamma_{U}$ is observable state, $d_{E}$, $d_{N}$ and $\sin A\gamma_{E}+\cos A\gamma_{N}$ become observable states based on ${Ν}_{3}x(t)$, and $d_{U}$ also becomes observable state based on ${Ν}_{4}x(t)$. As ${Ν}_{5}x(t)$ does not provide new state combinations, the rank of $Q_{sub}$ is the same as the rank of ${\left[\begin{array}{cccc} {Ν}_{1} & {Ν}_{2} & {Ν}_{3} & {Ν}_{4} \end{array}\right]}^{T}$, which is 10.

By applying the following transformation from the navigation frame to the sensor frame, we can obtain the sensor biases in the sensor frame as shown in Equation (24). As $\sin A\gamma_{E}+\cos A\gamma_{N}$ is observable state, $\gamma_{Y}$ and $\gamma_{Z}$ also becomes observable, while $\gamma_{X}$ cannot be uniquely determined. The gyro biases in X, Y and Z axis are observable states, because they are observable in the navigation frame:

(24) $$\left[\begin{array}{c} \gamma_{X}\\ \gamma_{Y}\\ \gamma_{Z} \end{array}\right]=\left[\begin{array}{c} \cos A\gamma_{E}-\sin A\gamma_{N}\\ \cos\omega t[\cos p(\sin A\gamma_{E}+\cos A\gamma_{N})-\sin p\gamma_{U}]+\sin\omega t[\sin p(\sin A\gamma_{E}+\cos A\gamma_{N})+\cos p\gamma_{U}]\\ -\sin\omega t[\cos p(\sin A\gamma_{E}+\cos A\gamma_{N})-\sin p\gamma_{U}]+\cos\omega t[\sin p(\sin A\gamma_{E}+\cos A\gamma_{N})+\cos p\gamma_{U}] \end{array}\right]$$

When roll has a non-zero value, R and $R^{2}$ can be calculated as follows. By examining the matrix, ${\left[\begin{array}{cccc} {Ν}_{2} & {Ν}_{3} & {Ν}_{4} & {Ν}_{5} \end{array}\right]}^{T}x(t)$, $\varepsilon_{E}$, $\varepsilon_{N}$, $\gamma_{E}$, $\gamma_{N}$, $d_{E}$, $d_{N}$, $d_{U}$ can be derived from the different combinations of states, and the rank of $Q_{sub}$ is 11.

(25) $$R=C_{b}^{n}\Omega_{bs}^{b}C_{n}^{b}=\omega \left[\begin{array}{ccc} 0 & c_{13}c_{22}-c_{12}c_{23} & c_{13}c_{32}-c_{12}c_{33}\\ c_{23}c_{12}-c_{22}c_{13} & 0 & c_{23}c_{32}-c_{22}c_{33}\\ c_{33}c_{12}-c_{32}c_{13} & c_{33}c_{22}-c_{32}c_{23} & 0 \end{array}\right]$$

(26) $$R^{2}=C_{b}^{n}\Omega_{bs}^{b}\Omega_{bs}^{b}C_{n}^{b}=\omega^{2}\left[\begin{array}{ccc} -c_{12}^{2}-c_{13}^{2} & -c_{12}c_{22}-c_{13}c_{23} & -c_{12}c_{32}-c_{13}c_{33}\\ -c_{22}c_{12}-c_{23}c_{13} & -c_{22}^{2}-c_{23}^{2} & -c_{22}c_{32}-c_{23}c_{33}\\ -c_{32}c_{12}-c_{33}c_{13} & -c_{32}c_{22}-c_{33}c_{23} & -c_{32}^{2}-c_{33}^{2} \end{array}\right]$$

where $C_{ij}$ is the element of $C_{b}^{n}$ at the ith row and jth column. □

(2) Continuous Rotation about Y axis

**Property** **4.** *When pitch is zero, the rank of* $Q_{sub}$ *is 10, and the observable states and combinations of states are:* $\delta v_{E}$*,* $\delta v_{N}$*,* $\delta v_{U}$*,* $g\varepsilon_{N}+\gamma_{E}$*,* $-g\varepsilon_{E}+\gamma_{N}$*,* $-\cos A\gamma_{E}+\sin A\gamma_{N}$*,* $\gamma_{U}$*,* $d_{E}$*,* $d_{N}$*,* $d_{U}$*; When pitch is not zero, the rank of* $Q_{sub}$ *is 11, and all error states become observable, except for azimuth error.*

**Proof** **4.** When pitch is zero, R and $R^{2}$ can be calculated as follows:

(27) $$R=C_{b}^{n}\Omega_{bs}^{b}C_{n}^{b}=\omega \left[\begin{array}{ccc} 0 & 0 & \cos A\\ 0 & 0 & -\sin A\\ -\cos A & \sin A & 0 \end{array}\right]$$

(28) $$R^{2}=C_{b}^{n}\Omega_{bs}^{b}\Omega_{bs}^{b}C_{n}^{b}=\omega^{2}\left[\begin{array}{ccc} -\cos^{2}A & \sin A\cos A & 0\\ \sin A\cos A & -\sin^{2}A & 0\\ 0 & 0 & -1 \end{array}\right]$$

By examining ${\left[\begin{array}{ccc} {Ν}_{3} & {Ν}_{4} & {Ν}_{5} \end{array}\right]}^{T}x(t)$, we can see that $d_{E}$, $d_{N}$ and $-\cos A\gamma_{E}+\sin A\gamma_{N}$ become observable states based on ${Ν}_{3}x(t)$, and $d_{U}$ becomes observable state based on ${Ν}_{4}x(t)$, the rows in ${Ν}_{5}$ are linear combinations of the rows of ${Ν}_{3}$ and the last row of ${Ν}_{4}$, so the rank of $Q_{sub}$ is same as the rank of ${\left[\begin{array}{cccc} {Ν}_{1} & {Ν}_{2} & {Ν}_{3} & {Ν}_{4} \end{array}\right]}^{T}$, which is 10. Similar to the IMU rotation about the X axis, by applying the transformation from the navigation frame to the sensor frame, we find that the accelerometer bias in the rotation axis (Y-axis) cannot be solely observable, whereas the bias in X and Z axis are observable error states. □

When pitch is not zero, R and $R^{2}$ can be calculated as shown in Equations (29) and (30). Similar to the rotation about the X axis, the error states, $\varepsilon_{E}$, $\varepsilon_{N}$, $\gamma_{E}$, $\gamma_{N}$, $d_{E}$, $d_{N}$ and $d_{U}$ can be derived from the different combinations of states obtained from ${\left[\begin{array}{cccc} {Ν}_{2} & {Ν}_{3} & {Ν}_{4} & {Ν}_{5} \end{array}\right]}^{T}x(t)$, and the rank of $Q_{sub}$ is 11:

(29) $$R=\omega \left[\begin{array}{ccc} 0 & -c_{13}c_{21}+c_{11}c_{23} & -c_{13}c_{31}+c_{11}c_{33}\\ -c_{23}c_{11}+c_{21}c_{13} & 0 & -c_{23}c_{31}+c_{21}c_{33}\\ -c_{33}c_{11}+c_{31}c_{13} & -c_{33}c_{21}+c_{31}c_{23} & 0 \end{array}\right]$$

(30) $$R^{2}=\omega^{2}\left[\begin{array}{ccc} -c_{11}^{2}-c_{13}^{2} & -c_{11}c_{21}-c_{13}c_{23} & -c_{11}c_{31}-c_{13}c_{33}\\ -c_{21}c_{11}-c_{23}c_{13} & -c_{21}^{2}-c_{23}^{2} & -c_{21}c_{31}-c_{23}c_{33}\\ -c_{31}c_{11}-c_{33}c_{13} & -c_{31}c_{21}-c_{33}c_{23} & -c_{31}^{2}-c_{33}^{2} \end{array}\right]$$

(3) Continuous Rotation about Z axis

**Property** **5.** *When both pitch and roll are zeros, the rank of* $Q_{sub}$ *is 8, and the observable states and combinations of states are:* $\delta v_{E}$*,* $\delta v_{N}$*,* $\delta v_{U}$*,* $g\varepsilon_{N}+\gamma_{E}$*,* $-g\varepsilon_{E}+\gamma_{N}$*,* $\gamma_{U}$*,* $\omega \gamma_{E}+gd_{E}$*,* $\omega \gamma_{N}+gd_{N}$*; when either pitch or roll is not zero, the rank of* $Q_{sub}$ *is 11, and all error states become observable, except for the azimuth error.*

**Proof** **5.** When both roll and pitch are zeros, R and $R^{2}$ can be calculated as follows:

(31) $$R=\omega \left[\begin{array}{ccc} 0 & -1 & 0\\ 1 & 0 & 0\\ 0 & 0 & 0 \end{array}\right]$$

(32) $$R^{2}=\omega^{2}\left[\begin{array}{ccc} -1 & 0 & 0\\ 0 & -1 & 0\\ 0 & 0 & 0 \end{array}\right]$$

By examining ${\left[\begin{array}{ccc} {Ν}_{3} & {Ν}_{4} & {Ν}_{5} \end{array}\right]}^{T}$, the rank of the matrix is 2, and its rows satisfy the following relations that:

(33) $$\begin{array}{l} {Ν}_{3}(1)={Ν}_{4}(2)=-{Ν}_{5}(1)\\ {Ν}_{3}(2)=-{Ν}_{4}(1)=-{Ν}_{5}(2)\\ {Ν}_{3}(3)={Ν}_{4}(3)={Ν}_{5}(3)=0_{1\times 12} \end{array}$$

where ${Ν}_{i}(k)$ is the kth row of matrix ${Ν}_{i}$. Therefore, the rank of $Q_{sub}$ is 8, and in addition to the observable states and combinations of states, $\delta v_{E}$, $\delta v_{N}$, $\delta v_{U}$, $g\varepsilon_{N}+\gamma_{E}$, $-g\varepsilon_{E}+\gamma_{N}$, $\gamma_{U}$, obtained from ${Ν}_{1}x(t)$ and ${Ν}_{2}x(t)$, the observable combinations of states, $\omega b_{E}+gd_{E}$ and $\omega b_{N}+gd_{N}$, can be obtained from ${Ν}_{3}x(t)$.

When pitch or roll is not zero, R and $R^{2}$ can be calculated as shown in Equations (34) and (35). Similar to the rotation about the X or Y axis, based on the different combinations of error states obtained from ${\left[\begin{array}{cccc} {Ν}_{2} & {Ν}_{3} & {Ν}_{4} & {Ν}_{5} \end{array}\right]}^{T}x(t)$, the roll and pitch errors, accelerometer biases in the east and north directions, as well as gyro biases become observable error states, and the rank of $Q_{sub}$ is 11:

(34) $$R=\omega \left[\begin{array}{ccc} 0 & c_{12}c_{21}-c_{11}c_{22} & c_{12}c_{31}-c_{11}c_{32}\\ c_{22}c_{11}-c_{21}c_{12} & 0 & c_{22}c_{31}-c_{21}c_{32}\\ c_{32}c_{11}-c_{31}c_{12} & c_{32}c_{21}-c_{31}c_{22} & 0 \end{array}\right]$$

(35) $$R^{2}=\omega^{2}\left[\begin{array}{ccc} -c_{11}^{2}-c_{12}^{2} & -c_{11}c_{21}-c_{12}c_{22} & -c_{11}c_{31}-c_{12}c_{32}\\ -c_{21}c_{11}-c_{22}c_{12} & -c_{21}^{2}-c_{22}^{2} & -c_{21}c_{31}-c_{22}c_{32}\\ -c_{31}c_{11}-c_{32}c_{12} & -c_{31}c_{21}-c_{32}c_{22} & -c_{31}^{2}-c_{32}^{2} \end{array}\right]$$

Based on the above analysis, we can see that the observability of the azimuth errors cannot be improved through IMU rotations. In fact, as the sensed earth rotation rate is swallowed by the random errors of MEMS gyros, the azimuth error is unobservable without maneuvers for the MEMS-based inertial system. In the presence of maneuvers, the horizontal velocity error model can be described by Equations (36) and (37) as follows:

(36) $$\delta {\dot{v}}_{E}=2\omega_{ie}\sin L\delta v_{N}-2\omega_{ie}\cos L\delta v_{U}+g\varepsilon_{N}-f_{N}\varepsilon_{U}+\gamma_{E}$$

(37) $$\delta {\dot{v}}_{N}=-2\omega_{ie}\sin L\delta v_{E}-g\varepsilon_{E}+f_{E}\varepsilon_{U}+\gamma_{N}$$

where $f_{N}$ and $f_{E}$ represent the accelerations in the north and east directions, respectively. Obviously, $F_{n}$ of the inertial error model in Equation (7) becomes $\left[\begin{array}{ccc} 0 & g & -f_{N}\\ -g & 0 & f_{E}\\ f_{N} & -f_{E} & 0 \end{array}\right]$. According to Equation (20), we can obtain the new observable error state combinations, $g\varepsilon_{N}-f_{N}\varepsilon_{U}+\gamma_{E}$ and $-g\varepsilon_{E}+f_{E}\varepsilon_{U}+\gamma_{N}$, when maneuvers are present. As $\varepsilon_{E},\varepsilon_{N},\gamma_{E}$ and $\gamma_{N}$ are observable states with IMU rotations, the azimuth error, $\varepsilon_{U}$, can be estimated naturally, and then the observability matrix becomes full rank, which makes the inertial system fully observable. □

## 4. Tests and Results Analysis

**Figure 2:**
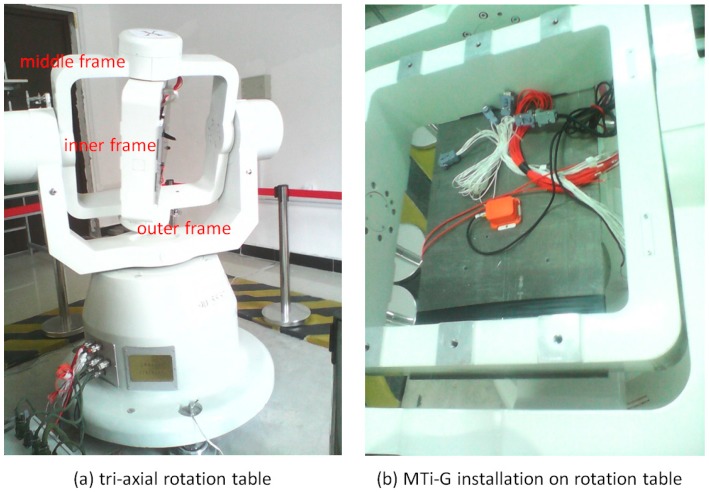
Tri-axial rotation table and MTi-G installation. (**a**) tri-axial rotation table; (**b**) MTi-G installation or rotation table.

Tests are conducted using a tri-axial rotation table with MTi-G to verify the improvements on the system observability by IMU rotations. The tri-axial rotation table has three rotational frames, namely, outer frame, middle frame and inner frame, as shown in the Figure 2a. A console controls the position and rotation of these frames. An initialization process, after which both middle and inner frames are in the level position and the rotation axis of inner frame points to north direction, is required for the rotation table. The MEMS IMUs are firmly installed on a piece of metal underneath the inner frame by screws, as shown in the Figure 2b. Different rotation schemes, such as rotation about X, Y and Z axes can be implemented by rotating the three frames. The MTi-G is a low-cost MEMS IMU produced by Xsens, and its error parameters can be found in [35].

Both non-rotating and rotating tests are conducted to verify the improvements on the observability of an inertial system when the maneuvers are absent. For the non-rotating test, both the rotation table and MEMS IMU remain still, while the IMU is rotating along with the rotation table in the rotating tests. IMU rotation about the Y and Z axes are tested, as the improvements by rotation about the X axis are similar to the ones by rotation about the Y axis. The IMU data is collected with a data rate of 100 Hz, while the rotated angle of the rotation table is collected at 50 Hz. As the rotation of IMU does not bring linear movements, the Zero Velocity Update (ZUPT) [37] is applied to estimate the attitude errors and inertial sensor errors in the tests using a KF. The filter state vector includes the velocity and attitude errors in the navigation frame, as well as the gyro and accelerometer biases in the body frame or sensor frame. The sensor biases are modeled as 1st Gauss-Markov random process, the model parameters are obtained by conducting an autocorrelation analysis for the collected data from the static inertial sensors [36].

### 4.1. Results Analysis for IMU Rotation about Y axis

**Figure 3:**
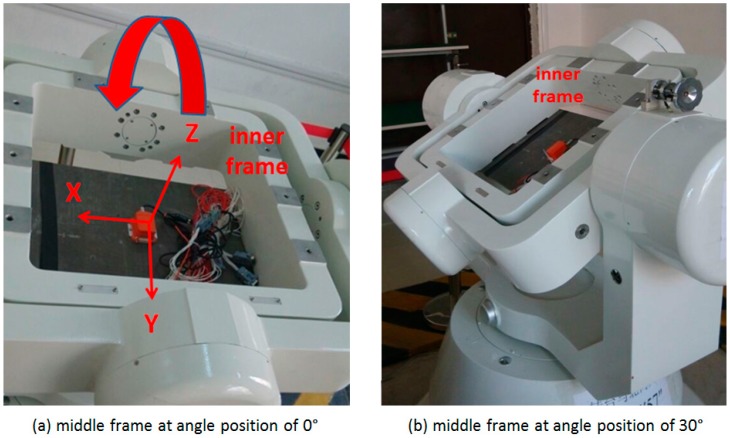
Rotation table set-up for IMU rotation about Y axis.

With the IMU sensitive axes defined as the X axis pointing right, the Y axis pointing forward, and the Z axis pointing up, as shown in the plot (a) of Figure 3, the rotation of the inner frame rotates the IMU about its Y axis when both the middle and outer frames remain still. Two rotating tests are conducted with the middle frame remaining at angle positions of 0° (level position) and 30°, respectively, to study the effect of the pitch angle on the system observability, as shown in the plot (a) and (b) of Figure 3. After the initialization process, the initial attitude (roll, pitch and azimuth) is 0°, 0° and 0°, when the middle frame remaining at the angle position of 0°; while the initial attitude is 0°, 30° and 0°, when it remains at the angle position of 30°. For each test, the IMU rotates about the Y axis with a constant rate of 10°/s.

When the roll, pitch and azimuth are 0°, 0° and 0°, respectively, the sensor biases in X, Y and Z axes are the same as the sensor biases in the east, north and up directions, respectively, for the non-rotating system; while for the rotating system, the relationships between the sensor biases in the sensor frame and the navigation frame can be described by as follows. The sensor bias in the Y axis is same as the bias in the north direction, while the sensor biases in the east and up directions are determined by the sensor biases in the X and Z axes, and modulated to periodic signals:

(38) $$\left[\begin{array}{c} \nabla_{E}\\ \nabla_{N}\\ \nabla_{U} \end{array}\right]=\left[\begin{array}{ccc} \cos\omega t & 0 & -\sin\omega t\\ 0 & 1 & 0\\ \sin\omega t & 0 & \cos\omega t \end{array}\right]\left[\begin{array}{c} \nabla_{X}\\ \nabla_{Y}\\ \nabla_{Z} \end{array}\right]=\left[\begin{array}{c} \nabla_{X}\cos\omega t-\nabla_{Z}\sin\omega t\\ \nabla_{Y}\\ \nabla_{X}\sin\omega t+\nabla_{Z}\cos\omega t \end{array}\right]$$

**Figure 4:**
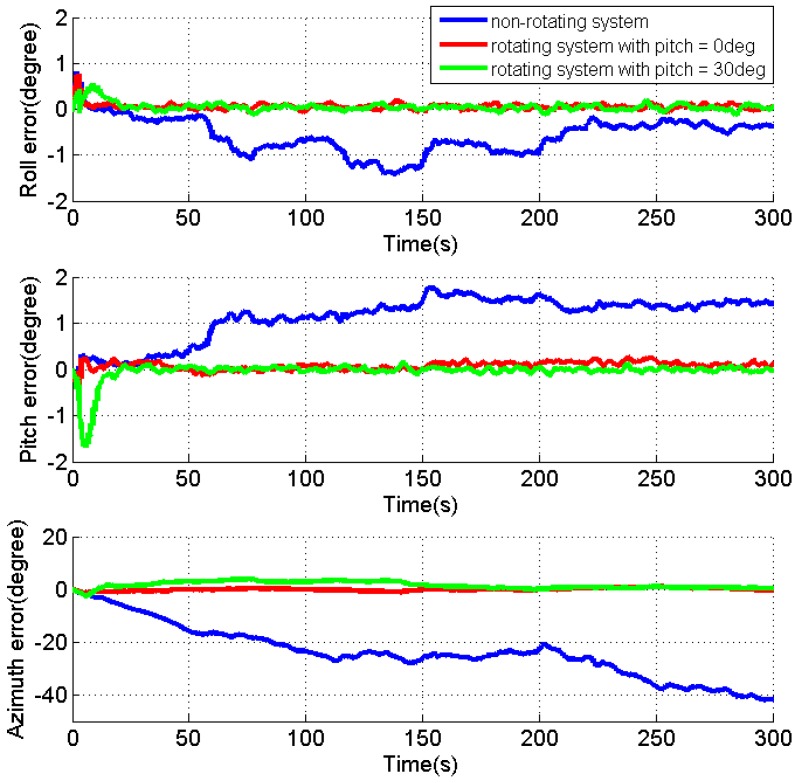
Estimates of the attitude errors for the non-rotating test and rotating tests with IMU rotation about the Y axis.

**Figure 5:**
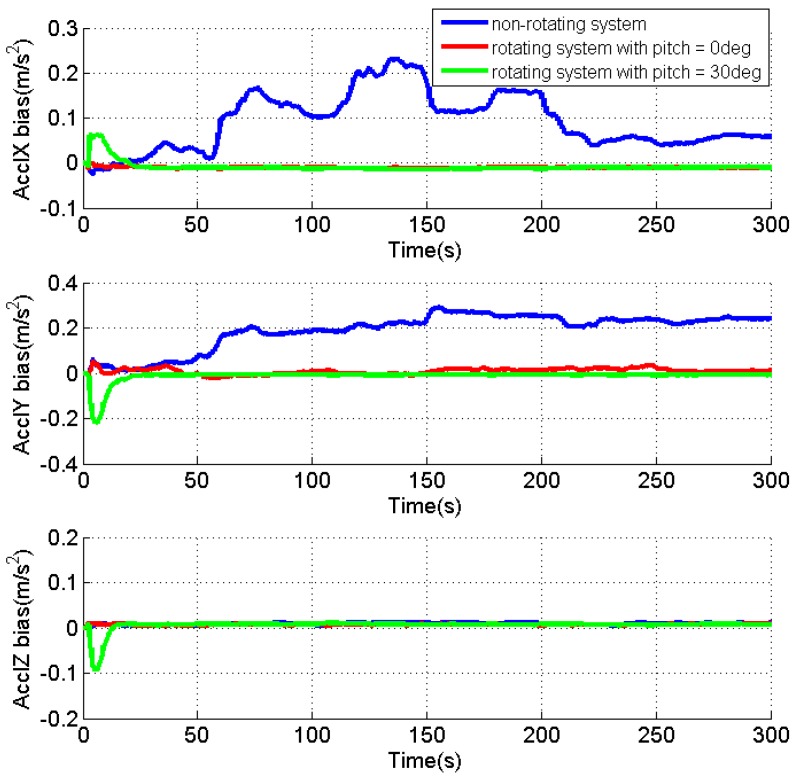
Estimates of the accelerometer biases for the non-rotating test and rotating tests with IMU rotation about the Y axis.

**Figure 6:**
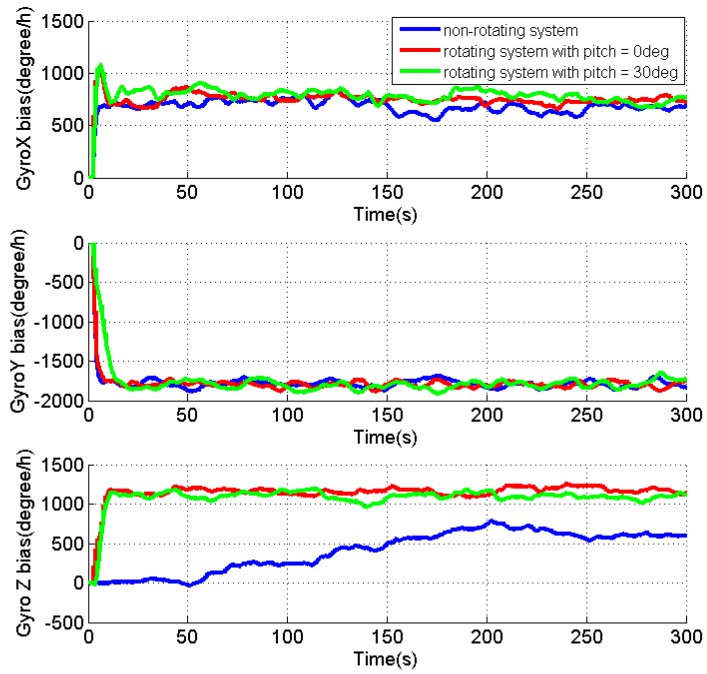
Estimates of the gyro biases for the non-rotating test and rotating tests with IMU rotation about Y the axis.

As the velocity errors can be directly observed, the analysis is focused on the attitude errors and inertial sensor biases. Figure 4, Figure 5 and Figure 6 present the estimates of the attitude errors, the accelerometer and gyro biases in the X, Y and Z axes, respectively. For the non-rotating system, we can obtain the following findings according to Property 1 when the roll, pitch and azimuth are 0°, 0° and 0°, respectively: (1) only $\gamma_{Z},d_{X},d_{Y}$ are observable error states; (2) the pitch and roll errors are jointly observable with $\gamma_{Y}$ and $\gamma_{X}$, respectively; (3) the azimuth error and $d_{Z}$ are unobservable. This is consistent with the obtained results that (1) the estimates of $\gamma_{Z},d_{X},d_{Y}$ are quickly converged as they are observable error states; (2) the estimates of the roll and pitch errors, as well as $\gamma_{Y}$ and $\gamma_{X}$ cannot converge, as they are jointly observable; (3) unconverged estimates of $d_{Z}$ leads to fast accumulation of the azimuth error.

According to the Property 4, we can obtain the following findings for the rotating system with the same attitude: (1) $\gamma_{X},\gamma_{Z},\varepsilon_{N},d_{X},d_{Y},d_{Z}$ are the observable error states; (2) $\gamma_{Y}$ is jointly observable with the pitch error, $\varepsilon_{E}$; (3) the azimuth error is unobservable. The obtained results also prove the above findings that (1) the converged estimates are obtained for the sensor biases, $\gamma_{X},\gamma_{Z},d_{X},d_{Y},d_{Z}$, and the estimate of the roll error is quickly reduced to around zero; (2) the fluctuations observed in the estimates of $\gamma_{Y}$ and the pitch error indicate their reduced estimability due to the fact that the two errors are jointly observable; (3) although the azimuth error cannot be observed, the estimation of $d_{X}$ and $d_{Z}$ significantly reduces its accumulations.

Apparently, the obtained results also verify that all error states, except for the azimuth error, become observable in the rotating system when the pitch is 30°, according to Property 4.

Table 1 summarizes the RMS of the attitude errors and the estimates of the accelerometer and gyro biases. Comparing to the results for the non-rotating test, the attitude errors are significantly reduced when IMU is rotating, which indicate the enhanced estimability of the sensor biases, although there is no reference.

In summary, the IMU rotation about the Y axis improves the system observability that all error states, except for the azimuth error, become observable when pitch is not zero. Even though the azimuth error is still unobservable, the estimation of the gyro biases effectively limits its accumulation. The estimability of the accelerometer bias in the Y axis is reduced when pitch is zero, as the accelerometer biases in the east and north directions are jointly observable with roll and pitch errors in such condition. Although only the results for IMU rotation about the Y axis are present, similar results can be obtained for IMU rotation about the X axis. The only difference is that the roll angle of zero will reduce the estimability of accelerometer bias in the X axis.

### 4.2. Results Analysis for IMU Rotation about Z axis

**Figure 7:**
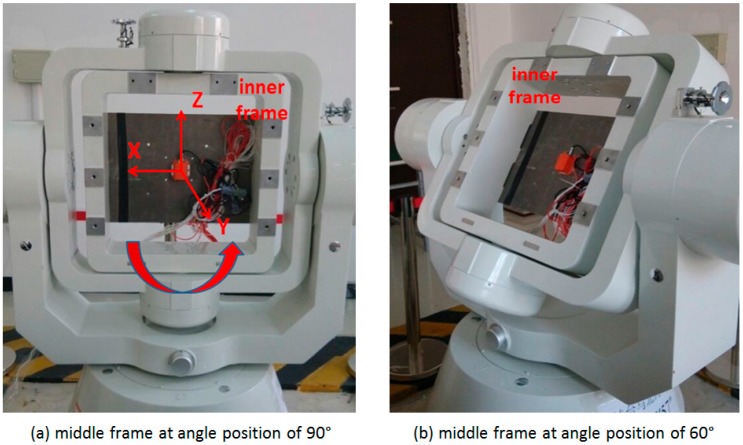
Rotation table set-up for IMU rotation about Z axis.

For the implementation of the IMU rotation about the Z axis, the middle frame of the rotation table is rotated to vertical position (90°), and the IMU axes are re-defined as the X axis pointing left, the Y axis pointing forward and the Z axis pointing up, as shown in the plot (a) of Figure 5. Then the rotation of the inner frame rotates the IMU about its Z axis. To verify the effect of the tilt angle on the system observability, two rotating tests are conducted with middle frame remaining at angle positions of 90° and 60°, respectively, as shown in the plot (a) and (b) of Figure 7. 

After the initialization process, the initial attitude is 0°, 0° and 0°, when the middle frame remaining at the angle position of 90°, and the initial attitude is 0°, 30° and 0°, when it remains at the angle position of 60°. For each test, the IMU rotates about the Z axis with the rotation rate of 10°/s.

When the roll, pitch and azimuth are 0°, 0° and 0°, respectively, the relationships between the sensor biases in the sensor frame and the navigation frame can be described by Equation (39) for the rotating system with IMU rotation about the Z axis. The sensor bias in the Z axis is same as the bias in the up direction, while the biases in the east and north directions become periodic signals by modulating the sensor biases in the X and Y axes:

(39) $$\left[\begin{array}{c} \nabla_{E}\\ \nabla_{N}\\ \nabla_{U} \end{array}\right]=\left[\begin{array}{ccc} \cos\omega t & \sin\omega t & 0\\ -\sin\omega t & \cos\omega t & 0\\ 0 & 0 & 1 \end{array}\right]\left[\begin{array}{c} \nabla_{X}\\ \nabla_{Y}\\ \nabla_{Z} \end{array}\right]=\left[\begin{array}{c} \nabla_{X}\cos\omega t+\nabla_{Y}\sin\omega t\\ -\nabla_{X}\sin\omega t+\nabla_{Y}\cos\omega t\\ \nabla_{Z} \end{array}\right]$$

**Figure 8:**
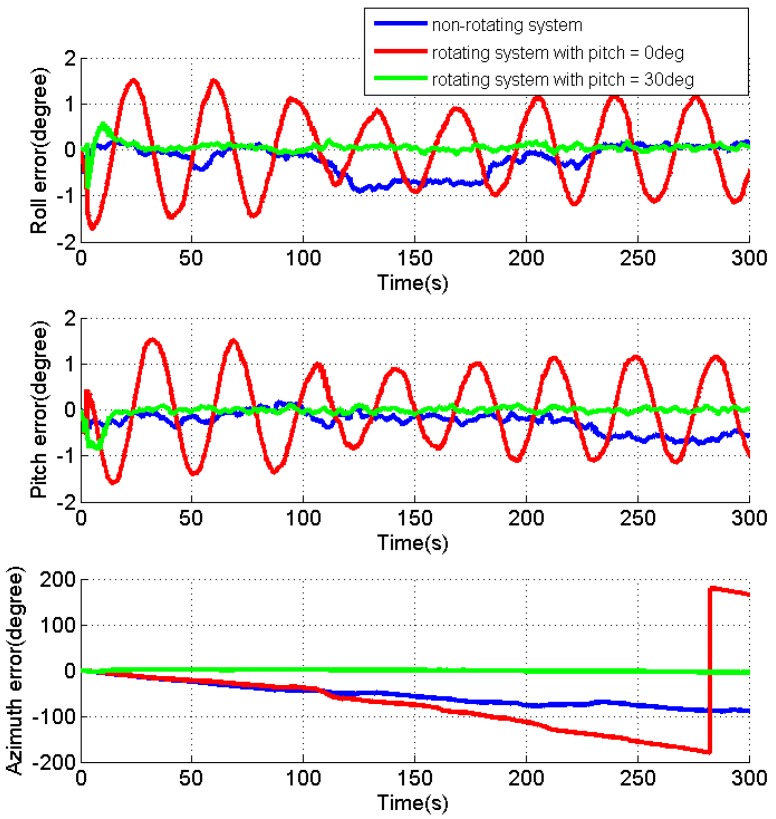
Estimates of the attitude errors for the non-rotating test and rotating tests with IMU rotation about the Z axis.

**Figure 9:**
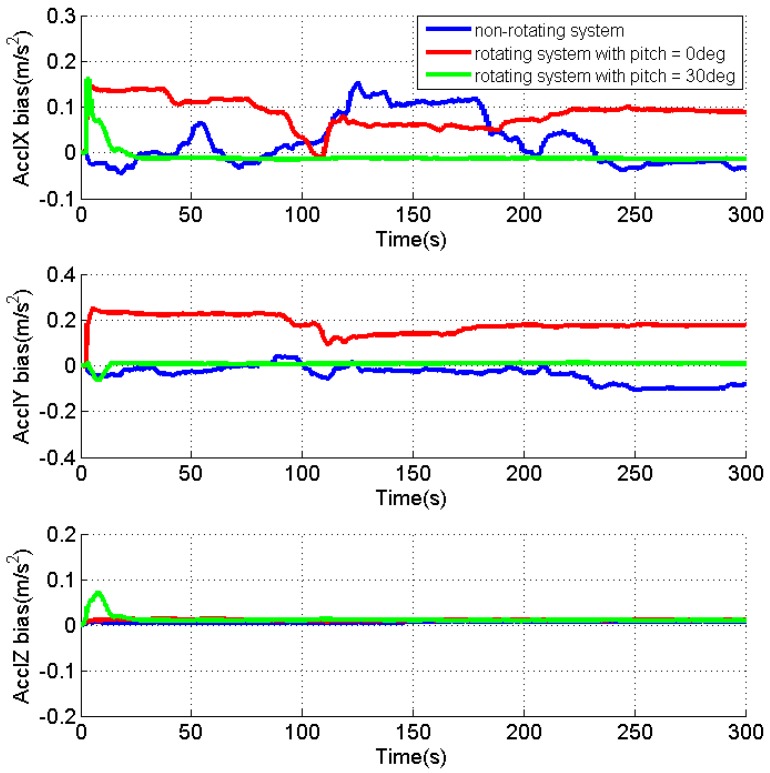
Estimates of the accelerometer biases for the non-rotating test and rotating tests with IMU rotation about the Z axis.

**Figure 10:**
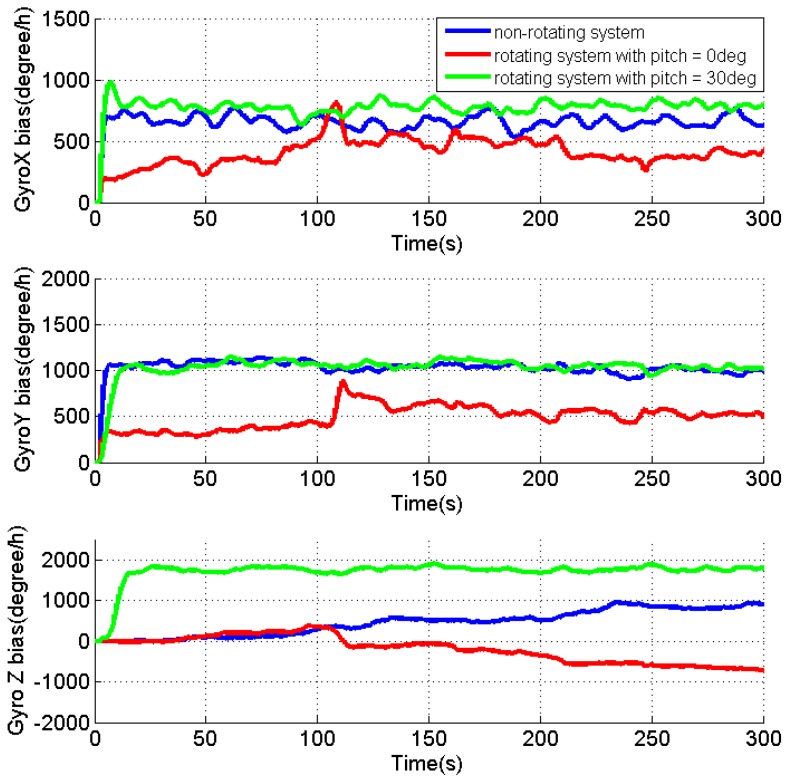
Estimates of the gyro biases for the non-rotating test and rotating tests with IMU rotation about the Z axis.

Figure 8, Figure 9 and Figure 10 present the estimates of the attitude errors, the accelerometer and gyro biases in X, Y and Z axes, respectively. According to Property 5, for the rotating system with the attitude of 0°, 0° and 0°, except for the velocity errors, $\gamma_{Z}$ is the only observable error state, and other jointly observable error states include $g\varepsilon_{N}+\gamma_{X}\cos\omega t+\gamma_{Y}\sin\omega t$, $-g\varepsilon_{E}-\gamma_{X}\sin\omega t+\gamma_{Y}\cos\omega t$, $\omega (\gamma_{X}\cos\omega t+\gamma_{Y}\sin\omega t)+g(d_{X}\cos\omega t+d_{Y}\sin\omega t)$ and $\omega (-\gamma_{X}\sin\omega t+\gamma_{Y}\cos\omega t)+g(-d_{X}\sin\omega t+d_{Y}\cos\omega t)$. As $\gamma_{X}$, $\gamma_{Y}$, $d_{X}$ and $d_{Y}$ can only be jointly observable, their estimates cannot be converged as shown in Figure 9 and Figure 10. Due to the failed estimation of those biases, the modulation of the bias residuals causes the oscillating roll and pitch errors as shown in Figure 8. The azimuth error is accumulated quickly as both the azimuth error and $d_{Z}$ are unobservable. When pitch is 30°, the estimates for the accelerometer and gyro biases in the X, Y and Z axes are quickly converged, while the roll and pitch errors are dropped to around zero. This verifies the Property 5 that when pitch is not zero, all error states (not including the azimuth error) become observable, and the enhanced estimation of $d_{Z}$ effectively limits the accumulation of the azimuth error.

Table 2 summarizes the RMS of the attitude errors, the estimated accelerometer and gyro biases. For the rotating system with a pitch angle, the improved observability of the sensor biases (the accelerometer biases in X and Y axes, as well as the gyro biases in the X, Y and Z axes) significantly reduce the attitude errors comparing to the results for the rotating system with pitch of 0°. Although there is no reference, the reduced attitude errors can verify the estimated sensor biases in the rotating system with a tilt angle.

Different from the IMU rotation about the X or Y axis, the tilt angle plays a very important role for the system with IMU rotation about the Z axis. Although the IMU rotation significantly improves the system observability with a tilt angle, it degrades the system observability when both the roll and pitch are zeros, comparing to the non-rotating system.

## 5. Conclusions

In the absence of vehicle maneuvers, the observable error states are the velocity errors, the accelerometer biases in the up direction, as well as the gyro biases in the east and north directions, for an inertial system based on a low-cost MEMS IMU. Other error states are only jointly observable, such as that the roll and pitch errors are jointly observable with the accelerometer biases in the east and north directions, which make them cannot be uniquely determined with velocity measurements. Moreover, the azimuth will be drifted quickly due to both the azimuth error and the gyro bias in the up direction are unobservable. This research employs the IMU rotation to improve the system observability. The observability analysis is conducted with a control-theoretic approach for the rotating system with IMU rotation about the X, Y and Z axis, respectively. Tests are also conducted to verify the improvements on the observability by IMU rotations. Both the theoretical analysis and results indicate that all error states, except the azimuth error, become observable through IMU rotations, and the enhanced estimability of the gyro bias in the up direction effectively limits the accumulation of the azimuth error. In fact, the system observability is also dependent upon the attitude. In certain conditions, the IMU rotation may degrade the observability of some error state that (1) the accelerometer in the X or Y axis cannot be solely observable with IMU rotation about the X or Y axis when the roll or pitch is zero, respectively; (2) the attitude errors, as well as the accelerometer and gyro biases, cannot be solely observable with IMU rotation about Z axis when both roll and pitch are zeros.

## Figures and Tables

**Table 1:** Estimates summary for non-rotating test and rotating tests with IMU rotation about Y axis.

	Roll (°)	Pitch (°)	Azimuth (°)	Accl X (m/s^2^)	Accl Y (m/s^2^)	Accl Z (m/s^2^)	Gyro X (°/h)	Gyro Y (°/h)	Gyro Z (°/h)
Non-rotating	0.71	1.24	25.91	0.112	0.223	0.009	683.1	−1786.7	495.9
Rotating (p = 0°)	0.07	0.11	0.78	−0.010	0.007	0.007	748.2	−1802.1	1165.2
Rotating (p = 30°)	0.08	0.08	2.02	−0.011	−0.007	0.008	777.1	−1804.9	1096.7

**Table 2:** Estimates summary for non-rotating test and rotating tests with IMU rotation about Z axis.

	Roll (°)	Pitch (°)	Azimuth (°)	Accl X (m/s^2^)	Accl Y (m/s^2^)	Accl Z (m/s^2^)	Gyro X (°/h)	Gyro Y (°/h)	Gyro Z (°/h)
Non-rotating	0.39	0.34	59.7	0.033	−0.039	0.006	653.6	1028.1	538.3
Rotating (p = 0°)	0.82	0.85	101.13	0.076	0.170	0.011	442.9	531.6	−243.4
Rotating (p = 30°)	0.08	0.08	2.96	−0.012	0.008	0.010	776.1	1056.4	1766.0
